# When knowing the activity is not enough to predict gaze

**DOI:** 10.1167/jov.24.7.6

**Published:** 2024-07-10

**Authors:** Andrea Ghiani, Daan Amelink, Eli Brenner, Ignace T. C. Hooge, Roy S. Hessels

**Affiliations:** 1 Department of Movement Sciences, Vrije Universiteit Amsterdam, Amsterdam, The Netherlands; 2 Department of Experimental Psychology, Utrecht University, Utrecht, The Netherlands

**Keywords:** gaze behavior, daily life activities, gaze–action coupling

## Abstract

It is reasonable to assume that where people look in the world is largely determined by what they are doing. The reasoning is that the activity determines where it is useful to look at each moment in time. Assuming that it is vital to accurately judge the positions of the steps when navigating a staircase, it is surprising that people differ a lot in the extent to which they look at the steps. Apparently, some people consider the accuracy of peripheral vision, predictability of the step size, and feeling the edges of the steps with their feet to be good enough. If so, occluding part of the view of the staircase and making it more important to place one's feet gently might make it more beneficial to look directly at the steps before stepping onto them, so that people will more consistently look at many steps. We tested this idea by asking people to walk on staircases, either with or without a tray with two cups of water on it. When carrying the tray, people walked more slowly, but they shifted their gaze across steps in much the same way as they did when walking without the tray. They did not look at more steps. There was a clear positive correlation between the fraction of steps that people looked at when walking with and without the tray. Thus, the variability in the extent to which people look at the steps persists when one makes walking on the staircase more challenging.

## Introduction

Many activities of daily life can be described in terms of sequences of actions (Botvinick & Plaut, 2004; Cooper & Shallice, 2000, Cooper & Shallice, 2006). The relation between such sequential actions and gaze behavior has been studied for activities such as walking in various environments, making tea and sandwiches, pouring water, and assembling a tent (Foulsham, Walker, & Kingstone, 2011; Ghiani, Mann, & Brenner, 2024; Ghiani, Van Hout, Driessen, & Brenner, 2023; Hessels, van Doorn, Benjamins, Holleman, & Hooge, 2020; Jovancevic, Sullivan, & Hayhoe, 2006; Jovancevic-Misic & Hayhoe, 2009; Matthis, Yates, & Hayhoe, 2018; Matthis & Fajen, 2014; Scrafton, Stainer, & Tatler, 2019; Sullivan, Ludwig, Damen, Mayol-Cuevas, & Gilchrist, 2021; Tatler, Hayhoe, Land, & Ballard, 2011). A common finding is that people shift their gaze toward items that are relevant for each of the sequential actions just before performing the actions (Hayhoe, 2000, Hayhoe, 2017; Hayhoe & Ballard, 2005, Hayhoe & Ballard, 2014). For instance, when preparing a cup of tea, gaze is directed toward the kettle and the cup just before grasping them (Land, Mennie, & Rusted, 1999). When making a sandwich, people look at the bread and at the knife just before interacting with them (Hayhoe, Shrivastava, Mruczek, & Pelz, 2003). This suggests that a comprehensive understanding of the sequence of actions that form an activity might make it possible to infer where and when people will look when performing that activity (Ballard & Hayhoe, 2009; Hayhoe, 2017; Hayhoe & Ballard, 2005, Hayhoe & Ballard, 2014). But is that always the case?

Some daily-life activities require one to monitor more locations in the visual world at the same time, so gaze has to regularly shift between these locations (Hayhoe & Ballard, 2005, Hayhoe & Ballard, 2014; Johnson, Sullivan, Hayhoe, & Ballard, 2014; Sprague & Ballard, 2003; Sullivan, Johnson, Rothkopf, Ballard, & Hayhoe, 2012). For example, if a driver decides to overtake a slower car ahead, the driver will have to regularly shift their gaze between the car driving in front of them and the image in the mirror as the time to change lanes approaches, to make sure to both maintain a safe distance to the car ahead and check that they are not currently being overtaken from behind (Johnson et al., 2014; Sullivan et al., 2012). Similarly, when walking in rough terrain, suitable locations to place one's foot have to be found for each step, so people need to frequently look where they are placing their foot. But they also need to occasionally look further ahead to plan their path, ensuring that there will be suitable positions to place their feet without hitting obstacles or losing balance further along the path (Marigold & Patla, 2007; Matthis et al., 2018; Matthis & Fajen, 2014; Patla & Vickers, 2003). It is probably difficult to predict precisely when people will switch where they look in such cases from an understanding of the sequence of actions that form the activities alone, but it might be possible if one considers the layout of the environment as well.

For other daily-life activities, one might not need to constantly monitor the situation at a specified location in the visual world, so people may have time to look at items in their surrounding that are not directly related to the actions they are performing. This can be the case for repetitive and predictable actions that do not require constant visual guidance. For example, in the study by Matthis and colleagues, when walking on smooth surfaces rather than on rough terrain, foot placement is less critical, so people look at the ground just in front of them less of the time (58% rather than 96% of the time), with more variability in the time spent looking at the ground just in front of them between participants (10% rather than 1%) (Matthis et al., 2018). At moments when one does not need to monitor a certain item or location, gaze behavior is likely to differ between people. In such cases, it may be more useful to know something about the person in question than to understand the sequence of actions that form the activity: While walking on a smooth path in a forest, bird watchers will probably look at different positions in the environment than mushrooms enthusiasts. Moreover, even when people do look where one would expect them to look for the activity they are performing, they may differ in terms of where precisely they look for various reasons, including social ones (Hessels, Benjamins, et al., 2020). Thus, the ability to infer where and when people will look when performing an activity probably depends on the extent to which where one looks matters for the activity, which is related to the predictability of the actions performed during that activity. This does not mean that visual information is not beneficial for such activities, but the extent and timing of when such information is needed is less fixed, so gaze behavior is likely to be less predictable from the task structure alone. It can therefore also differ considerably across individuals.

A good example of a repetitive and predictable activity where visual guidance is useful, but not necessary all the time, is stair climbing. Precise foot placement is critical on staircases (Zietz & Hollands, 2010), so we might expect people to look at steps before stepping onto them. Some studies found that people do so (Miyasike-DaSilva, Allard, & McIlroy, 2011; Zietz & Hollands, 2010), but others have demonstrated that looking at each step is not necessary (Den Otter, Hoogwerf, & Van Der Woude, 2011; Ioannidou, Hermens, & Hodgson, 2017; Miyasike-daSilva & McIlroy, 2012; Miyasike-daSilva, Singer, & McIlroy, 2019). For instance, Ioannidou and colleagues (2017) showed that people can successfully walk up staircases while typing messages on their phones rather than looking at the steps. Ghiani and colleagues (2023); Ghiani and colleagues (2024) found that when people are unaware that their gaze on staircases is being investigated, some look at most steps before stepping onto them, whereas others seldom look at steps. Even when descending staircases, which is more dangerous than ascending them, some people spend most of their time fixating other parts of the environment than the steps that they will step on. That people are able to walk up and down stairs without looking at the steps is probably because they can feel the steps with their feet, repeat previous equally sized strides, and guide foot placement with peripheral vision. Indeed, when peripheral vision is restricted, people tend to walk more carefully on staircases (Gimunová et al., 2018; Graci, Rabuffetti, Frigo, & Ferrarin, 2017). The use of peripheral vision in guiding actions has also been demonstrated in other tasks, such as driving, walking, and aviation (see Vater, Wolfe, & Rosenholtz, 2022, for a systematic review of these studies). Importantly, if there is no particular moment at which people have to look at the steps (Ghiani et al., 2023), it is evident that one cannot reliably predict where individual people will look at each moment as they walk up or down staircases, because people differ too much in when they look where.

We wondered whether we could make participants look at more steps, and thereby reduce the variability across participants, by partially obstructing visibility of the stairs, so participants could rely less on peripheral vision, and encouraging gentle and precise foot placement, which presumably makes it more beneficial to obtain precise visual information. To do so, we instructed participants to bring two trays, each with two glasses of water on them, to the starting location in a stairwell. One of the trays was originally on a higher floor and the other on a lower floor, so that participants walked both up and down staircases with and without a tray. The glasses of water made sure that the trays were held horizontally and that participants had to walk carefully to make sure not to spill any water. Holding the trays horizontally limited visibility of the stairs by occluding part of the lower visual field. We expected participants to look at more of the steps because they could rely less on seeing them while looking elsewhere and to do so more consistently because they had to step more carefully when they were carrying a tray.

## Method

### Participants

A total of 41 participants (age range: 18–32 years) with normal or corrected-to-normal vision and no physical impairments participated in the study. Before the experiment, participants received a brief explanation of the task and signed an informed consent form. The participants were aware that the experiment took place in a stairwell and that part of the task was climbing up and down staircases. Participants walked on their own and were completely unconstrained. The experiment was conducted in accordance with approval by the Scientific and Ethical Review Board of the Faculty of Behaviour and Movement Sciences of the Vrije Universiteit Amsterdam (file VCWE-2021-035).

### Task and data collection

Gaze was recorded with a Pupil Invisible eye tracker (recording frequency: 200 Hz), equipped with a camera (30 Hz; 1,088 × 1,080 pixels; 82° × 82° field of view) that recorded the visual scene in front of the participants. No audio was recorded. A description of the performance of the Pupil Invisible eye tracker can be found at arxiv:2009.00508 and in Hooge, Niehorster, Hessels, Benjamins, and Nyström (2022). The eye-tracking glasses were connected to a phone (OnePlus 8, Android version 11; Build 1: Oxygen OS 11.0.7.7.IN21IBA; Build 2: Oxygen OS 11.0.11.11.IN21BA) using a USB-c cable. The first nine participants were recorded while using Build 1. For comfort, the phone was secured in a small purse that could be worn around the neck.

**Figure 1.**
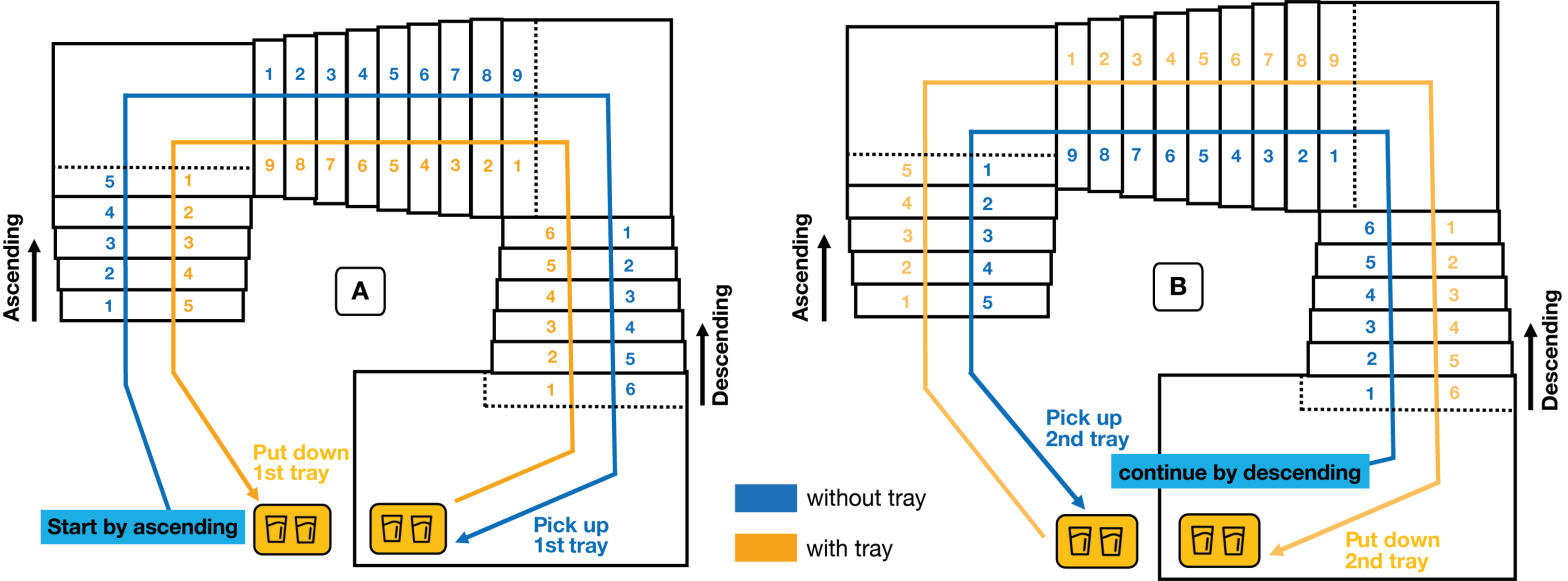
Walking route and experimental protocol. Participants started the experiment by ascending a flight of three staircases (**A**, with five, nine, and six steps, respectively) without a tray. They reached the upper floor, picked up the first tray with two glasses of water, and descended with the tray back to the starting point. Here, they put down the first tray and then descended a similar flight of staircases (**B**, with staircases of six, nine, and five steps, respectively) without a tray. When they reached the lower floor, they picked up the second tray with two glasses of water and went back up to the starting point, where they put down the second tray.

Before the experiment, participants performed a validation procedure to later assess the quality of the eye-tracking data (see section Eye-tracking data quality for details). After this phase, participants were guided to a nearby stairwell. All participants were familiar with this type of staircase. The steps were all equal in size and there were no objects on them. The experimenter positioned two serving trays (35 × 27 cm) at two predefined locations. Two paper cups filled to three fourths of their heights with water were placed on each of the trays. Participants were instructed to ascend a first flight of three staircases (with successively a five-, nine-, and six-step staircase; Figure 1A) to reach the upper floor (*without-tray* condition, ascending). Here, they picked up one of the trays and descended the same set of staircases with the tray until they reached the starting point, where they placed the tray on the floor (*with-tray* condition, descending). They then descended a new flight of three staircases (with successively six, nine, and five steps; Figure 1B) to reach the lower floor (*without-tray* condition, descending). Here, they picked up the second tray and ascended the same staircases back to the starting point, where they placed the second tray on the floor next to the first (*with-tray* condition, ascending). None of the participants spilled any water. All participants performed the task in the same order. This was done to avoid participants having to ascend two consecutive flights of staircases, which would have increased the probability of their getting fatigued. No instructions were given on how to carry the tray. The experimenter left the stairwell prior to the participant performing the task. Figure 1 shows the walking route for each condition.

### Data analysis

To compare gaze behavior during stair climbing with and without the tray, we computed the sequence in which gaze shifted across steps (as in Ghiani et al., 2023), the fraction of steps looked at, the fraction of looks on the tray and to the four sides of the tray, and the average time participants looked at a step.

**Figure 2.**
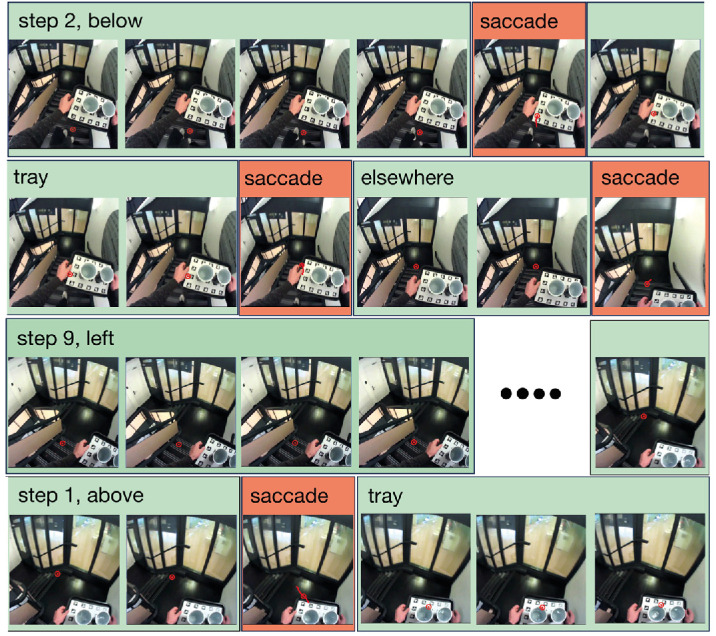
A frame-by-frame analysis was performed to label the fixated structures of interest. The images are consecutive frames from the video data, with gaze at the moment of the video frame indicated by the red circle, and all gaze measurements from half an interframe interval before to half an interframe interval after the current frame time between, indicated by the red path. The four dots indicate a jump of some frames. Red rectangles contain a saccade, as is evident from the length of the red path, and was automatically detected with a custom-built script. Each section indicated in green is assigned to a single label. In this example, looking at step 2 and step 9 was coupled with looking below and to the left of the tray, respectively. When looking at step 1, gaze could be considered both to be above and to the left of the tray. We decided to label these instances as above. Labels assigned to tray and elsewhere are also shown.

Once the parts of the data that contained the staircases were localized, a frame-by-frame analysis of the scene videos with gaze overlay was performed to label each item looked at, starting from the first fixation on any step and ending after the last fixation on any step. An item was considered to have been looked at if gaze was directed at about the same part of it for at least two frames of the scene camera video (about 66 ms). If gaze was only directed at it for one frame, it was not considered to have been looked at, but the eye was considered to have passed over it during a shift in gaze (see saccade frames in Figure 2). Thus, no label was assigned for such instances. Gaze was labeled either as looking at a step, in which case the step number was noted, or *elsewhere*. If a saccade displaced gaze on the same item (i.e., on step, tray, or elsewhere), the consecutive identical labels were merged into a single label, so sequential fixations on the same step were combined into a single step number as in Ghiani et al. (2023). For each participant and condition, this provided us with sequences of steps looked at, interleaved by periods of looking at the tray or elsewhere. Defining a look as one entry and exit of gaze from a region of interest (such as a particular step or the tray), irrespective of the duration and number of gaze shifts within the region, is similar to the definition of a “dwell,” as given in (Holmqvist et al., 2011, p. 190).

In addition to labeling gaze by the regions of interest, in the *with-tray* condition, we also determined the fraction of looks during which gaze was directed in various directions relative to the tray (above, below, left, or right), irrespective of whether gaze was directed at steps or elsewhere. When gaze was both above (or below) and to the left (or right) of the tray, we assigned it to above (or below), rather than left (or right), as in the *step 1, above* label in the bottom row of Figure 2.

To provide a general description of the sequence in which gaze shifted across the staircase, we examined how gaze transitioned between steps by computing the number of steps between pairs of successive looks at steps (*Direct* distribution). Shifting gaze to a step that will be reached later was considered positive (a shift from step 2 to step 5 would lead to a +3-step shift) and shifting gaze to a step that will be reached earlier negative (a shift from step 5 to step 2 would lead to a –3-step shift). Transitions were treated separately if gaze landed on the tray before shifting back to another step (*Indirect (tray)* distribution) or landed elsewhere before shifting back to one of the steps (*Indirect (elsewhere)* distribution). In these cases, there could also be *no steps* between pairs of successively steps looked at: Gaze could shift away from a step (either toward the tray or elsewhere) and then back to the same step. Occasionally, participants looked at both the tray and elsewhere between successive fixations on steps (3% of all indirect transitions when ascending and 10% when descending). In those cases, we arbitrarily attributed the step difference to *elsewhere*. It should be noted that this analysis is not a description of where people looked relative to their position on the staircase, as foot position is not taken into consideration. It is a description of how gaze shifted relative to the previous step that the participant looked at. We also measured the fraction of instances that each step was labeled, to check whether participants looked at the first and last few steps more frequently than they looked at other steps, which might be the case because that is when they cannot rely on the regularity of the steps.

From the labeled gaze data, we determined the fraction of steps that participants looked at by dividing the number of distinct steps of the three staircases that were looked at by the total number of steps in those staircases. This was done separately for each participant when ascending and when descending with and without a tray, giving us four values per participant. We used these values to determine whether the presence of the tray influenced the fraction of steps that were looked at and whether this was different when ascending and descending the staircases (repeated-measures analysis of variance with two factors: ascent or descent and with or without tray). This is our main measure, because we thought we could make participants look at more steps by asking them to walk with a tray.

Additionally, as descriptive measures, we determined the total time on the stairs for each participant as the time from when the foot was placed on the first step of the first staircase to when it was placed on the last step of the last staircase. This too was done separately for each condition (*without-tray* and *with-tray*) and direction (ascending and descending). The time point at which the foot was placed on a step was estimated from the output of the inertial measurement unit (IMU) in the eye tracker. We considered the time of the head's lowest position during each stride as the moment that the foot was placed stably (as in Ghiani at al., 2023). Within the same time period, we also computed the average time that gaze was directed toward a step by counting the number of frames in which the participant looked at the staircase for each condition and direction, and dividing this by the total number of steps fixated by that participant.

### Eye-tracking data quality

We present data quality measures based on the reporting guidelines by Dunn et al. (2023), reporting data loss, accuracy, and precision of the gaze position data. As a measure of data loss, we estimated the mean number of valid samples per second during the time the task was executed and report it as the effective frequency of the eye tracker (as suggested by Hooge et al., 2022). Accuracy and precision were estimated through a validation procedure performed by all participants before the experiment (Niehorster, Hessels, Benjamins, Nyström, & Hooge, 2023). While standing at arm's length from a nine-target validation poster, participants were asked to fixate on each target dot for about 1 second in reading order from the top left to the bottom right. In some cases, participants began fixating before receiving the full instructions or switched dots too quickly. In these instances, the entire validation procedure was repeated. We computed the mean angular distance between each target dot and the estimated gaze position when fixating that dot. Accuracy was defined as the mean of these angular distances across the nine targets. The root mean square (RMS) sample-to-sample deviation of the gaze position signal when looking at each target dot was also determined. Precision was defined as the mean RMS sample-to-sample deviation across the nine targets (Niehorster et al., 2023).

## Results

### Data quality

**Figure 3.**
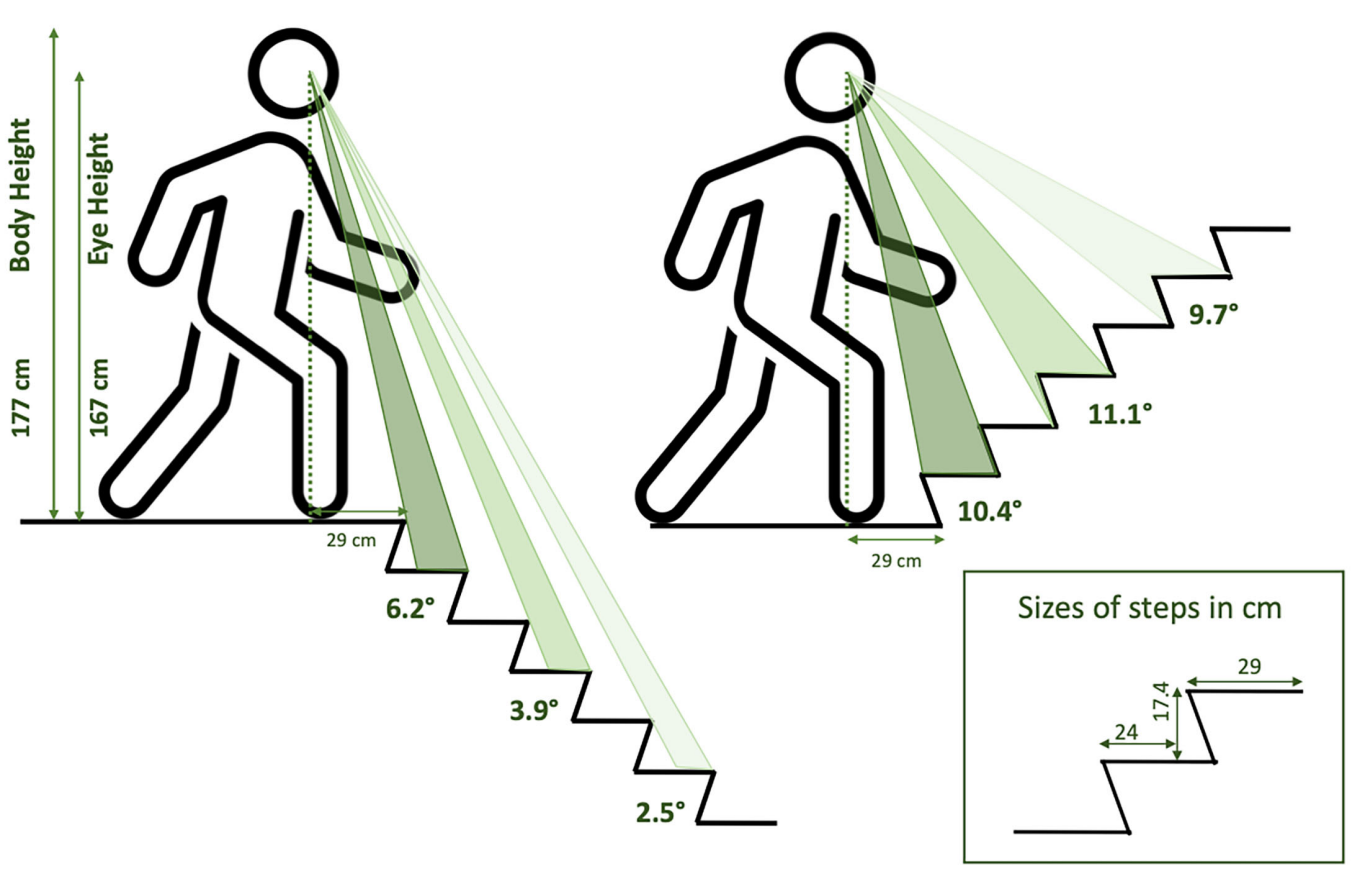
Visual angles covered by steps at three possible distances from the beginning of the staircase, as observed by a participant with a body height of 177 cm. The visual angles were computed separately for ascending and descending. Vertical and horizontal step dimensions are reported in the inset on the bottom right. Step width was between 117 and 120 cm.

The effective frequency of the eye tracker averaged across participants was 199.6 Hz (range across participants: 198.8–199.8 Hz). The overall accuracy averaged across targets and participants was 3.90° (range: 0.89°–9.64°). These values probably underestimate the accuracy because the gaze estimation method of the Pupil Invisible was presumably trained on images of eyes viewing objects at larger distances than arm’s length. This is consistent with an observed bias to the left in participants’ fixations of the targets on the validation poster (the scene camera is attached to the left side of the eye tracker). When looking at the stairs, gaze was obviously directed at items that were farther than arm’s length, so the leftward bias is presumably smaller than during the validation. The overall precision averaged across targets and participants was 0.19° (range: 0.09°–0.52°). To evaluate how the reliability of the gaze estimates might influence our results, we compared the estimated reliability to the angular dimensions of the steps at three distances from the participant when ascending and descending the staircases (Figure 3). These measures suggest that our data are accurate enough to assign gaze to the correct step when ascending staircases but that we may occasionally systematically be assigning gaze to a step above or below the one the participant is fixating when descending staircases. Note that this will seldom affect the estimated sequences across steps or the fraction of steps that are looked at. For example, if a participant looks at steps 1 and 3, this will lead to a *+*2 shift. Due to a systematic shift in the vertical direction, the actual labels could be assigned to steps 2 and 4 instead, still leading to a *+*2 shift.

### Gaze sequence

**Figure 4.**
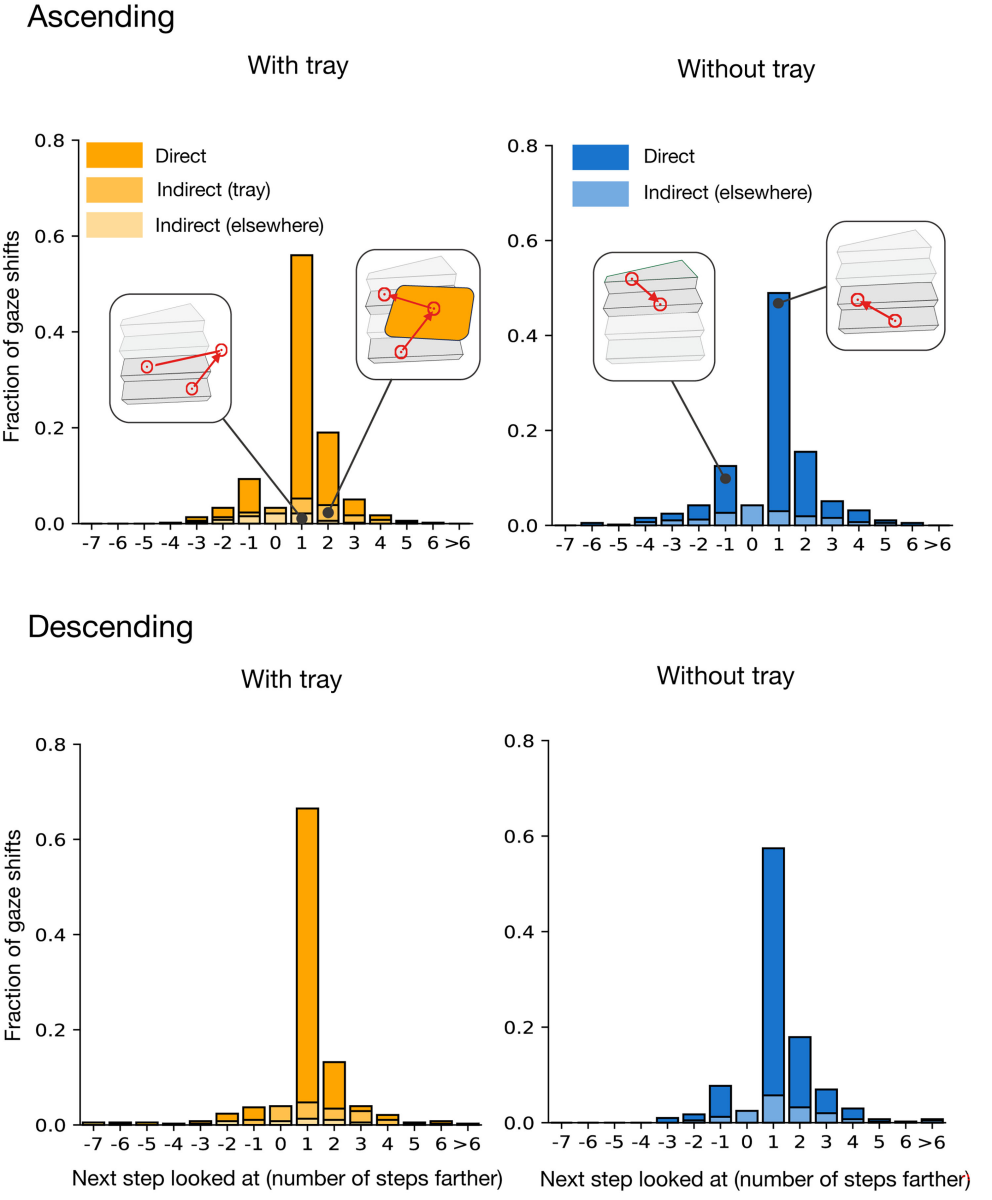
Frequency distribution of how many steps farther participants looked on the subsequent fixation when ascending (upper panels) and descending (lower panels) the staircases, separately for the *with-tray* condition (yellow) and *without-tray* condition (blue). *Direct* distributions (dark colors) show how many steps farther the participants looked when consecutive fixations were both on steps. *Indirect* distributions (light colors) show how many steps farther they looked after looking at the tray (*Indirect, tray*) or elsewhere (*Indirect, elsewhere*).

Participants did not shift their gaze differently across steps when carrying a tray compared to when not carrying a tray (Figure 4; *Direct*). The distribution of gaze shifts is also similar to what has previously been reported (Ghiani et al., 2023), showing a peak at *+*1 shifts. This makes sense when considering that participants keep walking forward. As expected, when carrying a tray (left panels of Figure 4), participants looked at the tray (*Indirect (tray)*, Ascending: 12%, Descending: 15%) more frequently than they looked elsewhere (*Indirect (elsewhere)*, Ascending: 8%, Descending: 6%), despite instances in which they looked both at the tray and elsewhere before looking at a next step being assigned to *elsewhere*. People looked elsewhere more frequently when not carrying the tray than when carrying it (*Indirect (elsewhere)*, Ascending: 18% rather than 8%, Descending: 17% rather than 6%).

### Fixated steps

**Figure 5.**
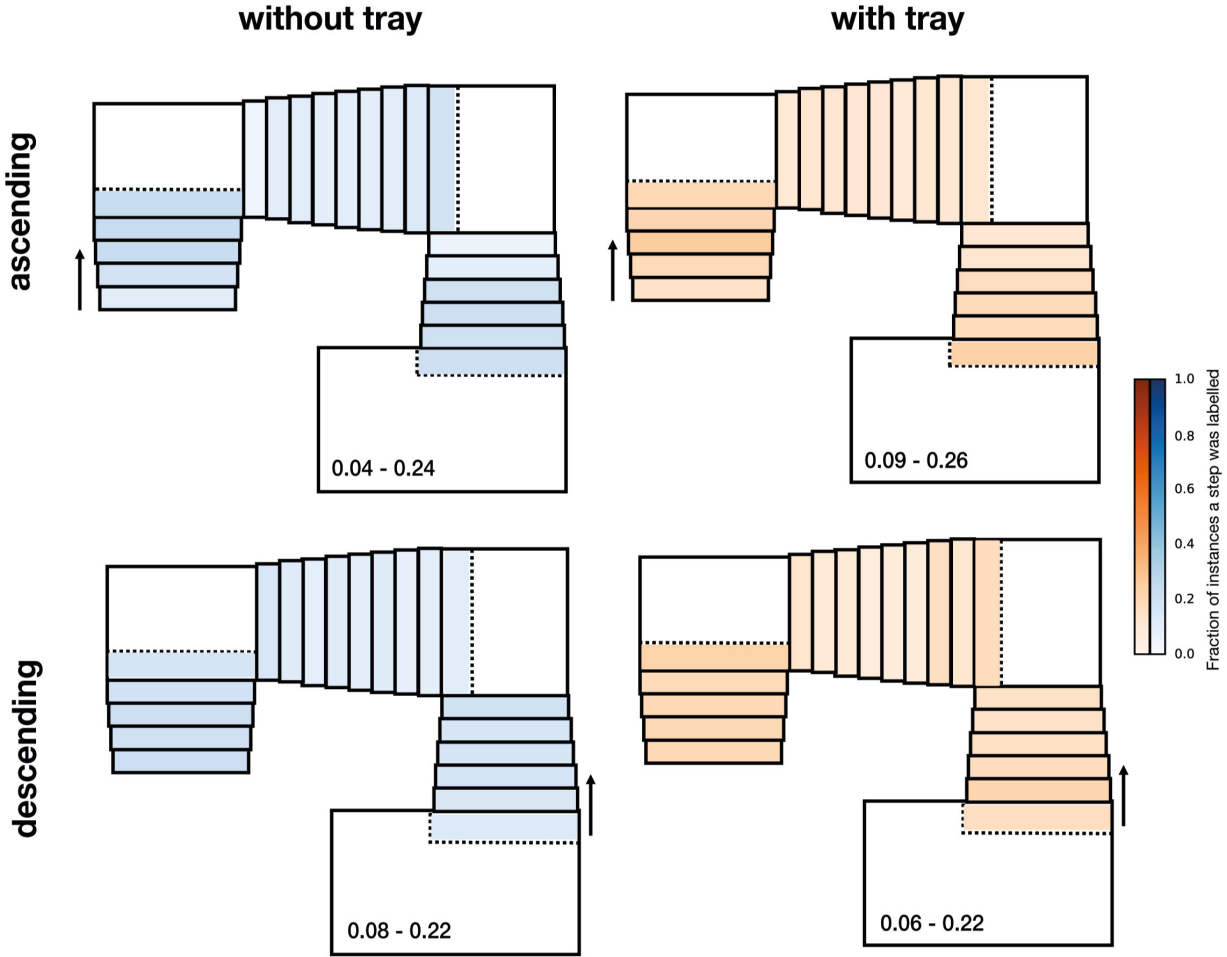
Fraction of instances each step was looked at, averaged across participants, when walking without a tray (blue) and with a tray (orange), both when ascending (upper panels) and descending (lower panels). Darker shades represent a higher fraction of instances that participants looked at the step. The range of fractions is indicated within each staircase.

Figure 5 shows that none of the steps were looked at particularly often. In particular, there was no tendency to preferentially look at the first and last steps, in line with earlier results showing that the first and last steps are not looked at more frequently than other steps when walking on staircases (Ghiani et al., 2023; Miyasike-DaSilva et al., 2011). Also quite surprisingly, but in accordance with earlier findings, there was even a tendency not to look at the first step when ascending.

### Fraction of steps looked at

**Figure 6.**
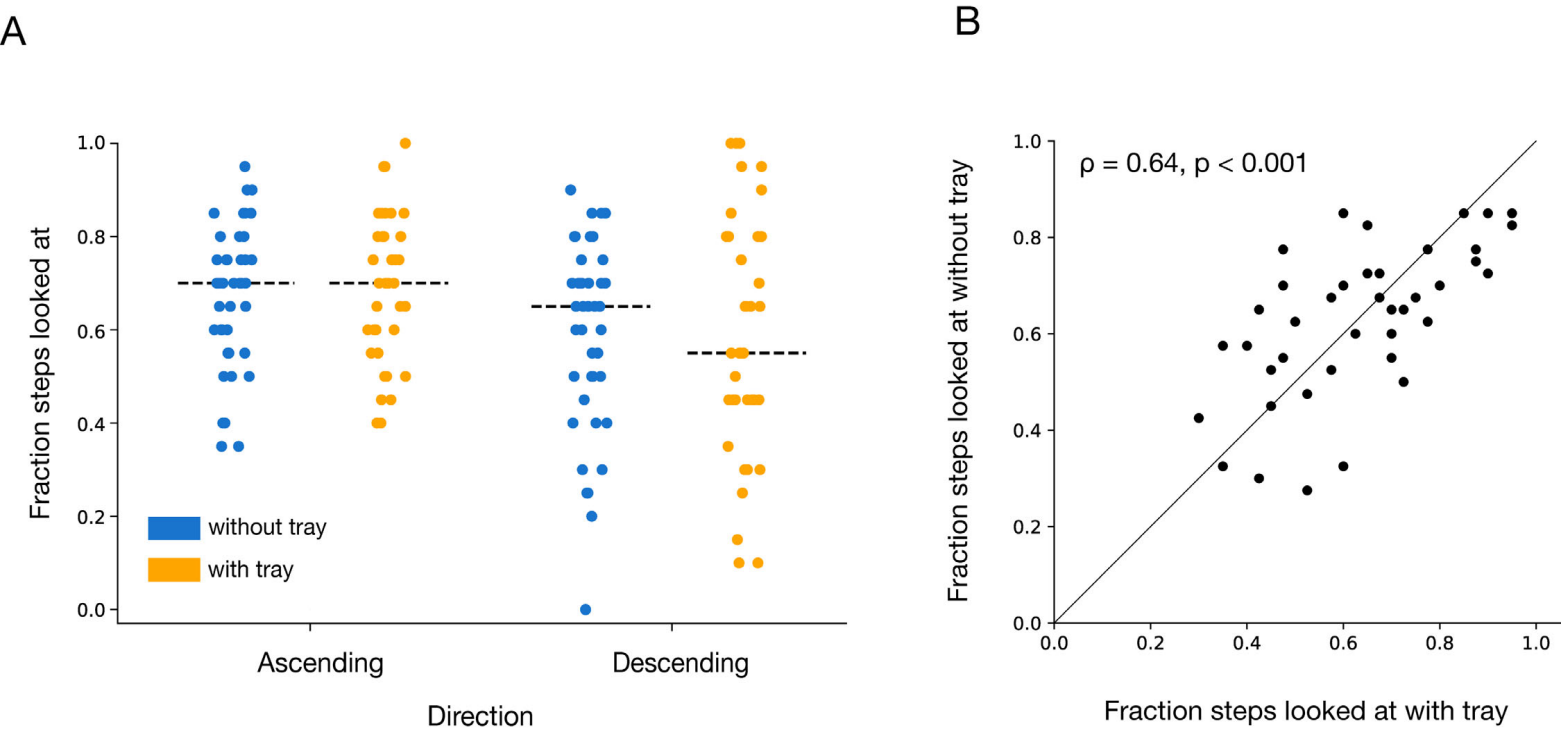
Fractions of steps looked at. (**A**) Individual participants’ values in the *with-tray* and *without-tray* condition when ascending and descending. The dashed lines show the median values. (**B**) Correlation between the fraction of steps looked at for the *with-tray* and *without-tray* condition, averaged across directions. Each dot represents one participant.

Contrary to our expectation, participants looked at a similar number of steps when walking with or without a tray, both when ascending and descending the staircases. Both without and with a tray, participants tended to look at more steps when ascending, as we have noted before (Ghiani et al., 2023). The analysis of variance with Direction (Ascending vs. Descending) and Condition (Without-tray vs. With-tray) as within-subjects factors revealed a significant main effect of Direction (*F*(1, 40) = 15.81; *p* < 0.001, *η*_p_^2^ = 0.28), but no significant main effect of Condition (*F*(1, 40) = 0.003; *p* = 0.95, *η*_p_^2^ = 0.00), or interaction between Condition and Direction (*F*(1, 40) = 0.58; *p* = 0.45, *η*_p_^2^ = 0.01) (Figure 6A). There was a significant positive correlation between participants’ fractions of steps looked at with and without the tray (Figure 6B). Thus, people who tended to look at many steps when not carrying the tray also looked at many steps when carrying the tray. This tendency is just as evident when examined separately for descending the staircases but is less clear when examined separately for ascending staircases. The lower correlation when ascending is likely due to there being less variability across participants when ascending (Figure 6A).

### Time on stairs

**Figure 7.**
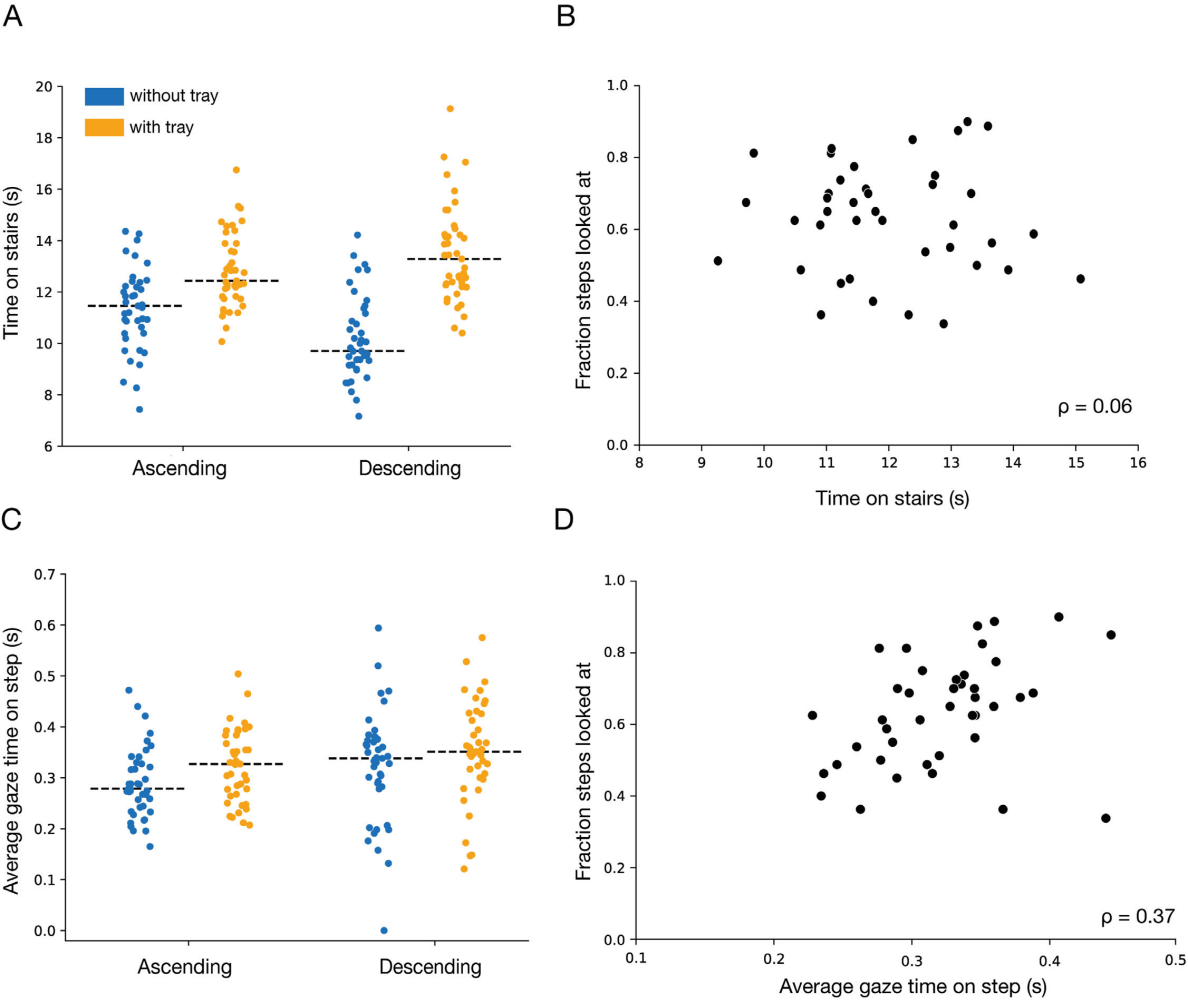
The total time spent on the stairs and the average time participants spent looking at a step that they looked at. (**A**) The total time that individual participants spent on the stairs in the *with-tray* and *without-tray* condition when ascending and descending. (**B**) Relationship between the total time on the stairs and the fraction of steps looked at. (**C**) Average time that individual participants spent looking at a step in the *with-tray* and *without-tray* condition when ascending and descending. (**D**) Relationship between the average gaze time spent on a step and the fraction of steps looked at. The dashed lines in **A** and **C** show the median values. In **B** and **D**, the values were averaged across directions (ascending, descending) and conditions (with tray, without tray).

Not surprisingly, people took more time to walk up and down the staircases when carrying a tray. The difference was particularly clear when descending (Figure 7A). In line with Ghiani et al. (2024), there was no indication that people who took more time looked at more steps (Figure 7B). There was also no such indication when considering the directions or conditions separately. In accordance with spending more time on the staircase (Figure 7A) but not looking at more steps (Figure 6A), participants might have looked at the steps that they did look at for slightly longer when carrying a tray (Figure 7C). Interestingly, we found a positive correlation between the average time spent looking at the steps that participants did look at and the fraction of fixated steps (Figure 7D), showing that participants who fixated steps longer also fixated more steps. Thus, the fraction of fixated steps does not appear to be limited by the total available time (in which case we would expect to see a positive correlation in Figure 7B) or the time taken to look at each step that one looks at (in which case we would expect to see a negative correlation in Figure 7D).

### Gaze positions relative to the tray

**Figure 8.**
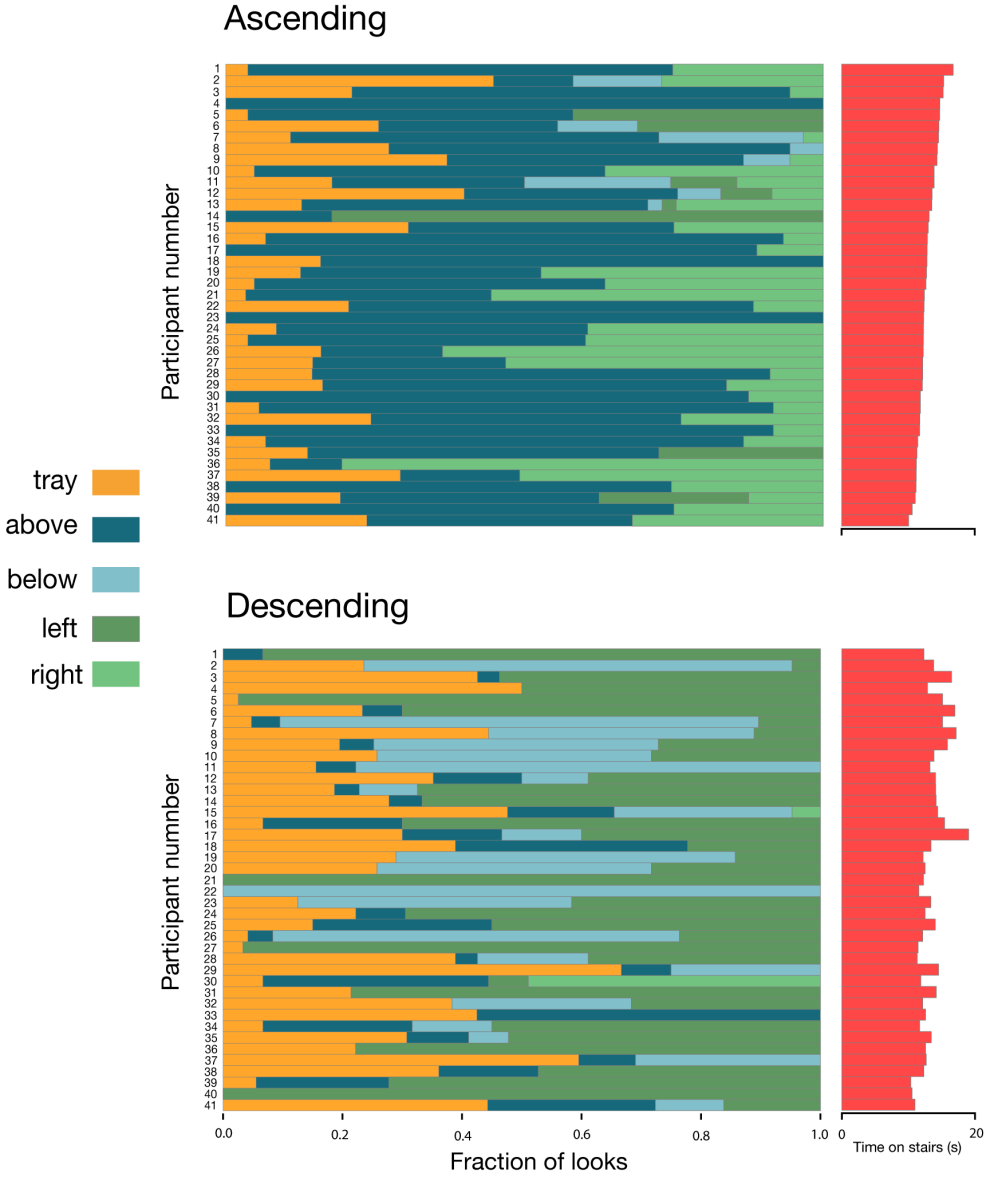
Fraction of looks on the tray or in the four directions relative to the tray (above, below, left, or right), separately for ascending (upper panel) and descending (lower panel). Each row is a participant. Participants are numbered according to their average overall time on the stairs when ascending, with participant 1 being the fastest.

When carrying a tray, participants differed from each other not only in the number of steps they looked at but also in how often they looked at the tray (Figure 8; participants are ordered by walking time from slowest to fastest when ascending, as shown by the bar plot on the right). Participants who walked the fastest (shortest times on stairs; represented in Figure 8 by high participant numbers) do not systematically have a lower fraction of looks on the tray. When ascending, people mostly looked above and to the right of the tray. When descending, they mostly looked to the left of and below the tray (Figure 8). Looking above the tray when ascending and below when descending is logical considering the geometry of the staircases (see Figure 3). The tendency to look to the right or to the left is probably related to the heading direction: When ascending with the tray, people had to turn right, while when descending, they had to turn left (Figure 1).

## Discussion

There are activities of daily life for which one can predict where people will look at various moments in time by knowing the activity alone (Ballard & Hayhoe, 2009; Hayhoe, 2017; Hayhoe & Ballard, 2005, Hayhoe & Ballard, 2014). However, this does not hold for all activities. Gaze during stair climbing has been shown to be highly variable across participants (Ghiani et al., 2023). The variability implies that it is impossible to predict where people will look at each moment in time for this activity, because where people look cannot be predicted by knowing the activity alone. We hypothesized that the reason for the large variability across participants is that for some participants, the task was so easy that they did not need to look anywhere on or near the stairs to successfully ascend or descend the staircase. Consequently, we anticipated that making the task more difficult would reduce the variability across participants by encouraging them to look at more steps. We made the task more difficult by having participants carry a tray with two glasses of water on them up and down the staircases. People did walk more slowly with the tray (Figure 7A), but contrary to our expectations, their gaze was very similar to that when walking without the tray, both in terms of the sequence of fixations (Figure 4) and of the number of steps they looked at (Figure 6).

Our findings are in line with the observation that participants take longer to walk a staircase when using a mobile phone but do not distribute their gaze differently when looking away from the phone: They showed similar dwell times on steps when walking with and without a phone (Ioannidou et al., 2017). The variability across participants in the fraction of fixated steps was not reported by previous studies on stair climbing, making it impossible to directly compare this aspect of their results with ours. That the variability was also large for related measures in some other studies can be inferred from the standard deviations reported in those studies, such as those for the number of fixations on stairs (Miyasike-DaSilva et al., 2011), and from individual participants’ data on the frequency of gaze shifting downward when descending (Miyasike-daSilva & McIlroy, 2016). These studies also used a lower sample size (10 and 11 vs. 41 participants in the current study) and measures more susceptible to noisy recordings, so it is difficult to say whether the reported standard deviation does represent actual variability across participants.

The only influence on the fraction of fixated steps that we found in this study was that participants looked at a higher fraction of steps when ascending compared to when descending. This is probably due to the staircase filling more of the field of view of the participant when ascending the staircase. Apparently, the staircase filling a larger part of the field of view has a larger influence on the number of steps that are fixated than the larger risks associated with falling when descending the staircase (Nagata, 1991; Ragg, Hwang, & Steinhart, 2000; Roys, 2001). Both this finding and the large variability in the fraction of fixated steps across participants are in line with our previous findings (Ghiani et al., 2023). This shows that the large variability in the number of fixated steps that we found in our previous study was not solely due to different staircases being used for every participant (in that study, people walked in their own home) but is at least partly due to systematic differences between individuals.

We can conclude from our study that examining the requirements of activities of daily life in sufficient detail is not always sufficient to predict people's gaze when performing such activities. This does not mean that there are no activities for which gaze is so critical that it becomes predictable. However, even for walking up and down stairs, for which vision is certainly important (although not necessarily critical at each moment), there is still a lot of room for idiosyncratic tendencies. Similarly, idiosyncratic differences have been shown in other daily-life tasks, such as driving, where peripheral vision is also important (Wiebel-Herboth, Krüger, & Wollstadt, 2021), and other domains, such as face perception, where idiosyncratic differences in face-scanning patterns have been found (Hessels, 2020; Hessels, Benjamins, et al., 2020; Mehoudar, Arizpe, Baker, & Yovel, 2014; Peterson, Lin, Zaun, & Kanwisher, 2016; Peterson & Eckstein, 2013).

We cannot ascertain whether the idiosyncratic differences arise from differences in the number of steps one needs to look at, or differences in how long one needs to look at the steps that one does look at, or differences in the wish to look elsewhere, but the correlation between participants’ behavior when walking with and without a tray (Figure 6B) indicates that it is not simply random variability. Moreover, as already mentioned, people who looked at more steps did not look at each step more briefly (on the contrary, they tended to look at them longer; Figure 7D) or walk more slowly so that they had more time to look at the steps (Figure 7B). Thus, participants slow down to step more gently so as not to spill any water, or because visibility of the stairs is partly occluded, rather than because walking slowly allows them to look at more steps.

We had assumed that participants would look at more steps when carrying a tray because looking at steps would be more beneficial. It would help to place one's feet precisely and gently, and compensate for the loss of peripheral vision of the staircase due to occlusion by the tray. However, looking at steps is more difficult when carrying a tray, and it may be beneficial to look at the tray to ensure that one keeps it horizontal, or to look at the cups on the tray to ensure that one does not spill any water. This might lead to fewer steps being fixated. Such opposite effects might even cancel each other in terms of the fraction of fixated steps. However, the consistency between the number of fixated steps when walking with and without a tray implies that balancing these considerations cannot be responsible for the large variability across participants.

We know that peripheral vision normally helps guide people's feet to steps on staircases, because people walk more cautiously when peripheral vision is occluded, especially when approaching the first steps and last step (Graci et al., 2017; Miyasike-daSilva et al., 2019). The fact that some participants consistently looked at few of the steps shows that the high acuity of central vision obtained by directing gaze to the steps is not crucial for the successful execution of this activity. This was even so when we occluded large parts of the peripheral visual field by making participants walk up and down the staircases carrying a tray with glasses of water on it. Partially occluding the stairs did not make people look at more steps, so they were presumably relying on other information. It is usually safe to assume that all steps have the same dimensions, so once the movement for one step is known, repeating the movement for upcoming steps may be enough to place the foot on the next step without directly looking at it. Moreover, once the foot is on the step, one receives feedback about the positioning from the contact itself, which can be used to guide the next foot placement (Cesini et al., 2020). Presumably, relying to a larger extent on such additional sources of information gives some people the freedom to look around more. Future research could investigate the actual influence of these sources of information on gaze behavior and whether differences in the number of fixated steps can be related to individual differences in the precision of or reliance on these sources.

A possible limitation of our study is the fixed order of conditions. This was done to reduce the likelihood of participants’ behavior being influenced by fatigue when ascending two consecutive staircases. The disadvantage of the fixed order is that participants might gradually become more familiar with the staircases. We doubt that this influenced our findings substantially, because all participants were already familiar with these staircases (they were all students at the university where data collection was performed). Moreover, we do not see systematically larger differences when comparing performance while participants were ascending the staircase, which were the conditions that were conducted first and last, than when they were descending the staircase, which were the conditions conducted in between.

The clear coupling between gaze and actions described for certain activities, such as tea making, has been often assumed to be “a common phenomenon in everyday life” (Land et al., 1999). It follows logically from the need to sample information throughout the action, especially in complex or dynamic environments in which relying on memory would be difficult. An association between gaze and the upcoming action is particularly evident for activities for which central visual guidance is needed, such as reading or tasks that require precision like hitting a nail and threading a needle. However, when considering more repetitive and predictable tasks, for which continuous information sampling through gaze is not strictly necessary, such as stair climbing, the gaze–action coupling is not so straightforward, even when trying to make looking at steps more beneficial. We believe that the absence of a strict coupling between gaze and actions is quite common in many activities of daily life that are characterized by repetitive and predictable patterns of action, such as walking, driving, cycling, running, and eating.
